# Factors associated with renal function state transitions: A population-based community survey in Taiwan

**DOI:** 10.3389/fpubh.2022.930798

**Published:** 2022-09-08

**Authors:** Ming-Hsien Tsai, Ming-Yen Lin, Chen-Yang Hsu, Amy Ming-Fang Yen, Tony Hsiu-Hsi Chen, Sherry Yueh-Hsia Chiu, Shang-Jyh Hwang

**Affiliations:** ^1^Division of Nephrology, Department of Internal Medicine, Shin-Kong Wu Ho-Su Memorial Hospital, Taipei, Taiwan; ^2^Division of Biostatistics, Institute of Epidemiology and Preventive Medicine, College of Public Health, National Taiwan University, Taipei, Taiwan; ^3^Master of Public Health Degree Program, College of Public Health, National Taiwan University, Taipei, Taiwan; ^4^Department of Renal Care, College of Medicine, Kaohsiung Medical University, Kaohsiung, Taiwan; ^5^Division of Nephrology, Department of Internal Medicine, Kaohsiung Medical University Hospital, Kaohsiung Medical University, Kaohsiung, Taiwan; ^6^School of Oral Hygiene, College of Oral Medicine, Taipei Medical University, Taipei, Taiwan; ^7^Department of Health Care Management, College of Management, Chang Gung University, Taoyuan, Taiwan; ^8^Department of Internal Medicine, Kaohsiung Chang Gung Memorial Hospital, Kaohsiung, Taiwan; ^9^Graduate Institute of Medicine, College of Medicine, Kaohsiung Medical University, Kaohsiung, Taiwan; ^10^Institute of Population Health Sciences, National Health Research Institutes, Zhunan, Taiwan

**Keywords:** chronic kidney disease, disease state, risk factor, illness-death model, risk prediction

## Abstract

**Background:**

Understanding renal function state transition risk and associated factors in community residences is vital for appropriate preventive and care actions. We aim to investigate factors affecting renal function state transitions through 10-year longitudinal community screening surveys.

**Methods:**

The prospective cohort study included participants who attended the screening program ≥2 times from 2001 to 2009 and were divided into two cohorts: those with baseline estimated glomerular filtration rate (eGFR) ≥60 (*n* = 46,278) and those with eGFR 59–30 mL/min/1.73 m^2^ (*n* = 4,656). We applied the illness-death model to identify associated factors with eGFR <60 and death for the cohort with baseline eGFR ≥60 and eGFR <30 and death for that with baseline eGFR ≥59–30.

**Results:**

Among the followed-up participants, 3,018 (6.5%) in the cohort of baseline eGFR ≥60 mL/min/1.73 m^2^ and 322 (6.9%) in the cohort of eGFR 59–30 mL/min/1.73 m^2^ experienced renal function state transition during a median over 7-year follow-up. Besides eGFR and grade of proteinuria, diabetes mellitus (adding nearly 50% hazard rate) is the main factor associated with both state transitions. Other early-phase eGFR state transition risk factors were metabolic syndrome score, triglyceride, uric acid, fasting blood sugar, and high-density lipoprotein cholesterol. Males, poor hemoglobin, high triglyceride, and high low-density lipoprotein cholesterol were all linked with the late-phase eGFR state transition hazard rate.

**Conclusion:**

The study developed the state transition functions for community participants with varying renal function levels. Further actions to develop precision screening plans and services that incorporate personal risk factors and state transition risks are necessary.

## Introduction

Chronic kidney disease (CKD) is a global public health problem accounting for 11.1% of the world population (1). CKD, without appropriate management, could rapidly progress to end-stage kidney disease (ESKD), requiring renal replacement therapy, which may incur substantial financial burdens to governments. According to the latest annual report of the United States Renal Data System, the annual ESKD prevalence (per million population) average yearly increased by 37.5% in reported countries from 2009 to 2018 (2); thus, seeking a sustainable development strategy to release these pressures is essential. Recent Kidney Disease: Improving Global Outcomes Controversies Conference emphasized the importance of early identifying risk stratification for renal function progression and delaying the time to advanced stages (3).

The annual average estimated glomerular filtration rate (eGFR) decline in healthy subjects is 0.97 ± 0.02 ml/min/1.73m^2^ per year (4) but could be more rapid when patients have different comorbidities and primary renal disorders (5). However, even for individuals with the same underlying cause of renal injury or degree of functional impairment, the eGFR declines could be highly variable. Early identification and management of people based on risks for CKD stage transition are essential worldwide. Previous epidemiological studies have reported that aristolochic acid digestion, diabetes, hypertension, proteinuria, hyperlipidemia, hyperuricemia, smoking habit, advanced age, male sex, and metabolic syndrome (MetS) were factors associated with CKD development and progression (6–9). Moreover, several CKD progression prediction models have been proposed (10–13), of which the endpoints were eGFR lower than 60 mL/min/1.73 m^2^, doubling of serum creatinine, 20% decrease in eGFR, and ESKD requiring renal replacement therapy. However, information on factors associated with state transition in renal function progression is quite a few especially in the community. The use of state transition as an endpoint can help healthcare providers easily communicate with patients and strengthen staged CKD prevention and management. Therefore, we analyzed longitudinal population-based community screening data to elucidate the factors influencing early- and late-phase eGFR state changes using the illness-death model.

## Methods

### Study design

A prospective cohort design was developed for participants aged ≥20 years, using a community-based multiple screening program in Keelung, the northernmost city of Taiwan. This screening program, the Keelung Community-based Integrated Screening (KCIS) program, was implemented from 1999 to 2009. The study used data from the screening results from 2001 to 2009 due to the lack of urine protein tests in the early 2 years. Details regarding the study design and implementation of this program prevalence have been reported elsewhere (14, 15). The CKD incidence and prevalence detected by the annual screening program have also been broadly noted (16). In brief, adults residing in Keelung and eligible for the KCIS program were invited annually for screening non-communicable diseases, such as cancers, diabetes, hypertension, dyslipidemia, and renal disease. However, participants may reattend the screening program at different year intervals. This program was governed by the Health Bureau of Keelung City, Taiwan, and approved by the local ethical committee of the Health Bureau of Keelung City. Written informed consent was received from all participants in each screening activity.

### Data collection

In the KCIS program, information was collected at each screening round, including data from a series of medical tests for blood, urine, body measurements, functional assessments, and medical history (questionnaire). The data also included anthropometric measures at the baseline, such as body mass index and waist circumference; biochemical variables, such as spot urine analysis using the dipstick method and serum creatinine, low-density lipoprotein cholesterol (LDL), high-density lipoprotein cholesterol (HDL), albumin, hemoglobin, uric acid, and fasting blood sugar levels; and lifestyle factors, such as alcohol intake, smoking, betel nut chewing, and physical activity. Participants were classified as drinkers and non-drinkers of alcohol, smokers and non-smokers, betel nut chewers and non-chewers, and regularly performing and not performing exercise according to drinking, smoking, betel nut chewing, and physical activity. Individual comorbidity included the presence of diabetes mellitus (DM), hypertension, and coronary artery disease (CAD). Moreover, proteinuria severity according to the spot urine dipstick analysis was recorded as follows: grade 0, absent; grade 1 (trace), 15–30 mg/dL; grade 2 (1+), 30–100 mg/dL; grade 3 (2+), 100–300 mg/dL; grade 4 (3+), 300–1,000 mg/dL; and grade 5 (4+), >1,000 mg/dL.

### Metabolic syndrome

MetS contains five components. It is defined according to the National Cholesterol Education Program Adult Treatment Panel III criteria (Expert Panel on Detection Evaluation and Treatment of High Blood Cholesterol In Adults, 2001) (17). MetS was defined when a participant exhibited more than three components which included the following: central obesity (waist circumference ≥80 cm for women and ≥90 cm for men), elevated blood pressure (systolic blood pressure ≥130 mmHg or diastolic blood pressure ≥85 mmHg), hyperglycemia (fasting glucose level ≥100 mg/dL), hypertriglyceridemia (serum triglyceride level ≥150 mg/dL), and low HDL (serum HDL level <50 mg/dL for women and <40 mg/dL for men). The MetS score was calculated as the sum of the scores for the presence of metabolic components.

### State of renal function decline and death

The eGFR values were calculated by age, sex, and serum creatinine through CKD-EPI equation (18). Participants who attended the KCIS program ≥2 times from 2001 to 2009 were categorized into the groups of eGFR ≥60 mL/min/1.73 m^2^ and eGFR 59–30 mL/min/1.73 m^2^ based on their values at the first screening. We defined the appearance of eGFR <60 as early-phase transition and <30 mL/min/1.73 m^2^ as late-phase transition at consequent screening visits for the groups of eGFR ≥60 mL/min/1.73 m^2^ and eGFR 59–30 mL/min/1.73 m^2^, retrospectively. Death between 2001 and 2010, ascertained by the National Death Registry of Taiwan, was considered an absorbed state.

### Statistical analysis

Baseline data are described using mean ± standard deviation or median with interquartile range (IQR) as appropriate for continuous variables and proportions for categorical variables. Because most of the exact time of eGFR state transition is unknown, we recorded age at the first screening visit, age at screening visit before eGFR state transition, age at screening visit after eGFR state transition, and age at death or the latest screening visit for both cohorts. The timing of any eGFR state transition between two subsequent screenings is usually unknown in almost all patients, and we considered the time to transition as interval-censored. Patients with CKD may be more likely to suffer from death than the eGFR stage transitions; we adopted a competing risk analysis with the illness-death model to fit the Weibull distribution. The illness-death model simultaneously estimates three-state (eGFR ≥60, eGFR <60, and death; eGFR 59–30, eGFR <30, and death) transition intensities by fitting Weibull regression functions using the Levenberg–Marquardt's algorithm to maximize the (penalized) likelihood (19, 20). This model firstly produces estimates for intensity and regression parameters. Then, through continuous estimates of intensity by the model could obtain relevant quantities, such as transition probabilities, cumulative probability, and life expectancies (21). To explore the relationship between continuous biochemical data and each eGFR transition, we categorized them by quartile and put the least quartile group as the reference group. All covariates besides age were forced entered to model development except the variable of betel nut chewing because of its small portion in the cohort with eGFR 59–30 mL/min/1.73 m^2^. We displayed model results by hazard ratio (HR) with 95% confidence intervals (CIs). Based on the developed models, we could apply the model to understand the risk of eGFR state transition based on participants' characteristics. The models predicted consequent annual transition probabilities by changing selecting participants' characteristics (male with DM, male without DM, female with DM, and female without DM) in a free participant for the defined state at 45 years old. The transition probability curves of the selecting participant's characteristics were compared to understand the participant's transition risks. A two-tailed *p* of <0.05 was considered statistically significant. All analyses were performed using R Studio (version 1.2.5042) with “SmoothHazard” packages (22) and SAS (version 9.4; SAS Institute Inc., Cary, NC, USA).

## Results

### Study participant characteristics

**Figure 1:**
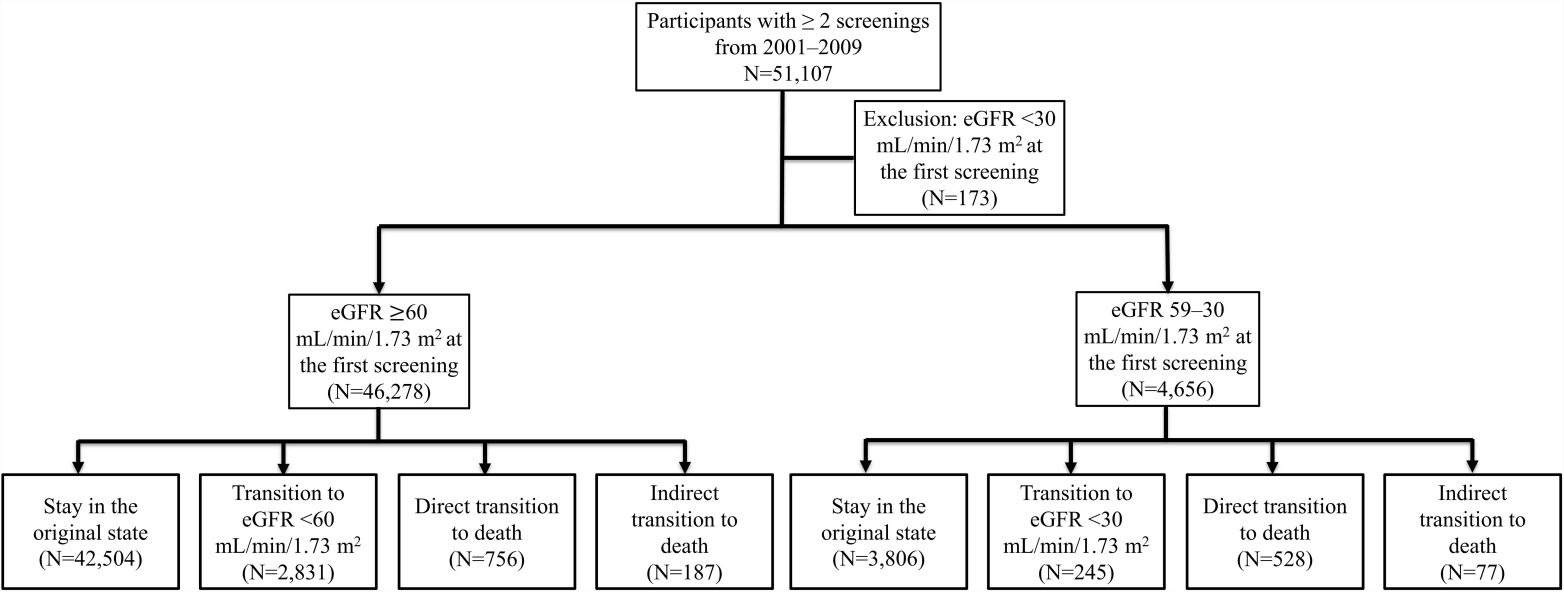
Study flowchart.

This study included 46,278 participants with eGFR ≥60 mL/min/1.73 m^2^ and 4,656 participants with eGFR 59–30 mL/min/1.73 m^2^. Of the observed participants, 3,018 (6.1%) in the group of eGFR ≥60 mL/min/1.73 m^2^ and 322 (6.9%) in the group of eGFR 59–30 mL/min/1.73 m^2^ experienced eGFR state transition during the follow-up period (Figure 1). The study population was slightly older and more female than the overall Keelung population (data not shown). The participant characteristics of our study cohorts are summarized in Table 1. Participants with eGFR 59–30 mL/min/1.73 m^2^ were significantly older (mean age, 68.2 ± 10.1 vs. 47.4 ± 13.5 years), more likely to be men (46.8 vs. 35.0%), and had DM (12.9 vs. 4.0%), hypertension (37.5 vs. 11.3%), and CAD (17.7 vs. 4.9%) than those with eGFR ≥60 mL/min/1.73 m^2^.

**Table 1:** Baseline characteristics of the study population.

**Variable**	**Estimated glomerular filtration rate, mL/min/1.73 m** ^**2**^	
	**≥60 (*n* = 46,278)**	**59–30 (*n* = 4,656)**
Age, y	47.4 ± 13.5	68.2 ± 10.1
Male sex	16,173 (35.0)	2,178 (46.8)
BMI (kg/m^2^)	24.2 ± 3.7	25.3 ± 3.6
Diabetes (%)	1,809 (4.0)	580 (12.9)
Hypertension (%)	5,133 (11.3)	1,713 (37.5)
CAD (%)	2,210 (4.9)	802 (17.7)
SBP (mmHg)	123.5 ± 19.4	135.9 ± 20.8
DBP (mmHg)	77.6 ± 11.9	80.6 ± 12.3
Waist circumference (cm)	78.1 ± 10.5	84.1 ± 10.0
Smoker (%)	10,788 (23.5)	1,170 (25.3)
Alcohol drinker (%)	9,978 (21.8)	847 (18.4)
Betel nut chewer (%)	2,359 (5.2)	81 (1.8)
Regular exerciser (%)	29,632 (65.5)	3,277 (71.5)
Metabolic syndrome (%)	8,582 (18.6)	1,688 (36.3)
Total cholesterol (mg/dL)	195.6 ± 37.9	208.4 ± 41.1
Triglyceride (mg/dL)	124.5 ± 116.0	150.5 ± 105.7
LDL (mg/dL)	113.4 ± 32.7	121.8 ± 35.2
HDL (mg/dL)	58.1 ± 14.2	57.2 ± 14.6
Glucose (mg/dL)	93.4 ± 24.6	102.1 ± 34.7
Hemoglobin (g/dL)	13.9 ± 5.8	13.7 ± 1.6
Albumin (mg/dL)	4.5 ± 0.3	4.4 ± 0.4
Uric acid (mg/dL)	5.6 ± 1.6	6.7 ± 1.9
Proteinuria
Grade 0 (%)	33,475 (88.9)	2,967 (71.0)
Grade 1 (%)	1,264 (3.4)	204 (4.9)
Grade 2 (%)	1,579 (4.2)	411 (9.8)
Grade 3 (%)	854 (2.3)	334 (8.0)
Grade 4 (%)	428 (1.1)	236 (5.6)
Grade 5 (%)	41 (0.1)	29 (0.7)
Events of state change (%)	3,017 (6.5)	321 (6.9)

BMI, body mass index; CAD, coronary artery disease; SBP, systolic blood pressure; DBP, diastolic blood pressure; HDL, high-density lipoprotein; LDL, low-density lipoprotein.

Sum of counts from different categories of one variable may not equal to overall n number due to missing values.

Conversion factors for units: total cholesterol, triglyceride, LDL in mg/dL to μmol/L × 0.02586, HDL in mg/dL to μmol/L × 0.02586, glucose in mg/dL to mmol/L × 0.05551, and uric acid in mg/dL to μmol/L × 59.48.

The estimated glomerular filtration rate value was calculated by the CKD-EPI equation.

### Age-specific baseline transition intensity

**Figure 2:**
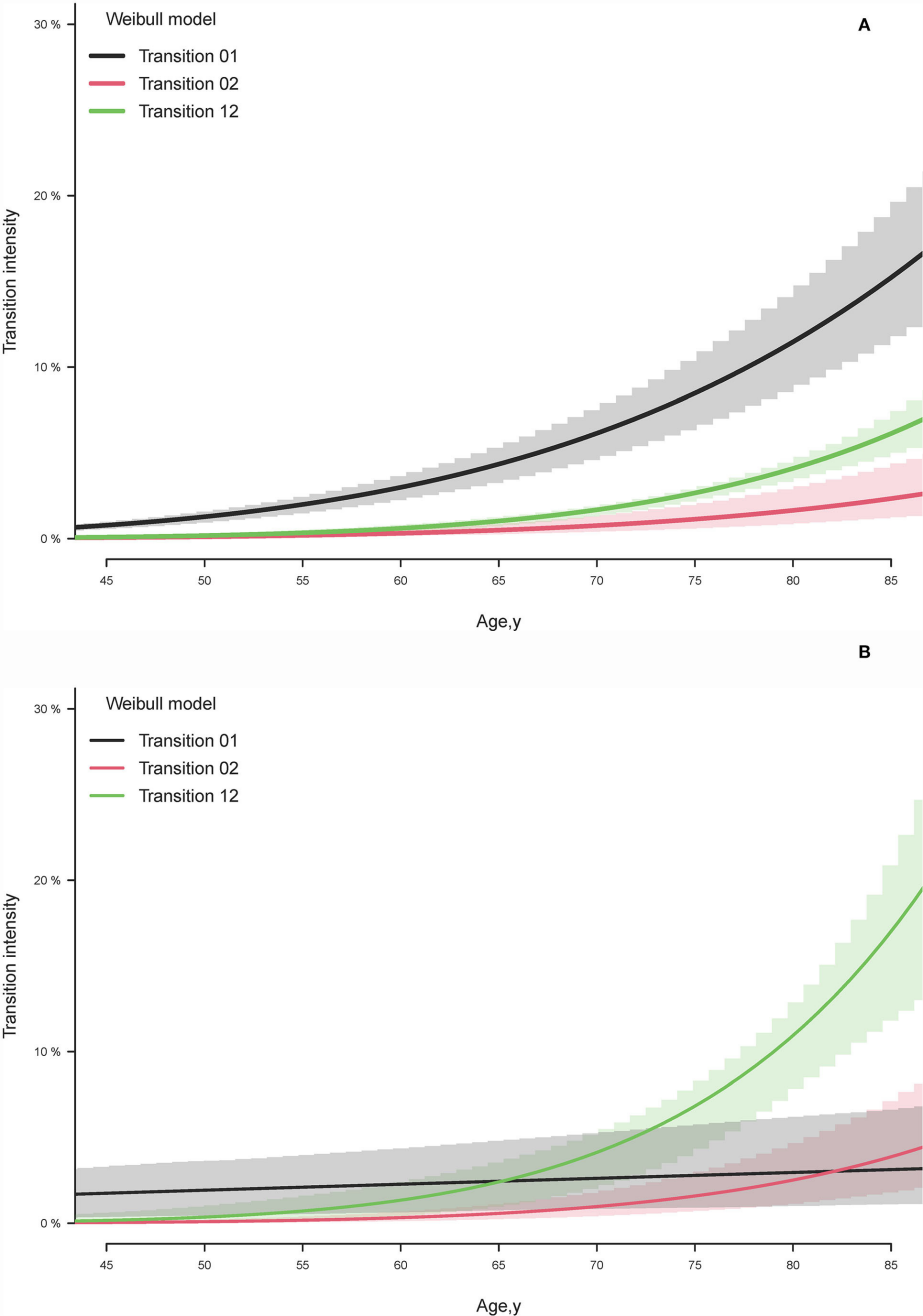
Estimated baseline transition intensity by illness-death model for **(A)** estimated glomerular filtration rate (eGFR) <60 state and death transitions in the cohort with baseline eGFR ≥60 ml/min/1.73 m^2^ and **(B)** eGFR <30 state and death transitions in the cohort with baseline eGFR 59–30 ml/min/1.73 m^2^. Transition 01 represents the transition intensity from the baseline state to the selected state. Transition 02 exhibits the transition intensity from the baseline state to death. Transition 12 displays the transition to death after transiting the selected state from the baseline state.

In the participants with eGFR ≥60 mL/min/1.73 m^2^, the model identified the curve relationship between baseline transition intensities and age (Figure 2A). The transition intensities of eGFR <60 are higher than the transition intensities of death across observed ages. The differences between transition intensities of eGFR <60 and death gradually increase with rising age, especially after 60 years. On the contrary, the age-specific baseline transition intensities of eGFR <30 in the participants with eGFR 59–30 are higher than death only before age 65 years (Figure 2B). The annual transition intensities of death directly from the baseline state rapidly raised in the cohort of eGFR 59–30 compared to eGFR ≥60, causing more than six times (19.4 vs. 3.0%) immediate risk for death at age 85 years.

### Factors associated with early-phase eGFR state and death transitions

The cohort with eGFR ≥60 mL/min/1.73 m^2^ was followed for a median time of 7.2 years (IQR, 5.3–8.3) and contributed a median-censored time of 4.0 years (IQR, 2.4–6.0). The significant factors associated with increased risk of early-phase eGFR state transition (transition to eGFR <60 mL/min/1.73 m^2^) were DM, MetS, rising quartile levels in triglyceride, uric acid, grad of proteinuria, and reducing quartile levels in fasting blood sugar, HDL, and eGFR (Table 2). In addition, being male sex, with CAD, a smoker, rising quartile levels in triglyceride, reduced quartile levels in hemoglobin, total cholesterol, triglyceride, and LDL were associated with an increased risk of death direct from eGFR ≥60 mL/min/1.73 m^2^.

**Table 2:** Factors associated with transiting to estimated glomerular filtration rate (eGFR) <60 mL/min/1.73 m^2^ or death in the cohort with baseline eGFR ≥60 mL/min/1.73 m^2^.

	**Transition to eGFR**<**60 mL/min/1.73 m**^**2**^		**Transition to death**	
	**Hazard ratio (95% CI)**	***p-*value**	**Hazard ratio (95% CI)**	***p-*value**
Sex				
Female	1.00 [Reference]		1.00 [Reference]	
Male	0.99 (0.86–1.15)	0.9	1.99 (1.44–2.74)	<0.001
Diabetes mellitus				
None	1.00 [Reference]		1.00 [Reference]	
Yes	1.50 (1.31–1.71)	<0.001	1.30 (0.90–1.89)	0.16
Coronary artery disease				
None	1.00 [Reference]		1.00 [Reference]	
Yes	1.09 (0.97–1.23)	0.16	1.57 (1.15–2.13)	0.005
Smoker				
None	1.00 [Reference]		1.00 [Reference]	
Yes	1.04 (0.93–1.17)	0.47	1.89 (1.44–2.49)	<0.001
Alcohol drinker				
None	1.00 [Reference]		1.00 [Reference]	
Yes	1.01 (0.89–1.14)	0.89	1.08 (0.83–1.41)	0.55
Betel nut chewer				
None	1.00 [Reference]		1.00 [Reference]	
Yes	1.11 (0.88–1.42)	0.38	1.39 (0.90–2.13)	0.13
Regular exerciser				
None	1.00 [Reference]		1.00 [Reference]	
Yes	1.02 (0.94–1.12)	0.61	0.84 (0.67–1.04)	0.11
Metabolic syndrome score	1.12 (1.06–1.17)	<0.001	1.00 (0.95–1.06)	0.9
Hemoglobin, g/dL				
Q1 (<12.8)	1.00 [Reference]		1.00 [Reference]	
Q2 (12.8–13.7)	1.00 (0.95–1.05)	0.9	0.78 (0.58–1.05)	0.10
Q3 (13.8–14.9)	0.94 (0.83–1.06)	0.29	0.65 (0.48–0.88)	0.006
Q4 (≥15)	1.00 (0.82–1.21)	0.9	0.64 (0.45–0.91)	0.01
Albumin, mg/dL				
Q1 (<4.3)	1.00 [Reference]		1.00 [Reference]	
Q2 (4.3–4.4)	1.03 (0.92–1.14)	0.65	1.11 (0.84–1.46)	0.46
Q3 (4.5–4.6)	1.02 (0.91–1.15)	0.72	0.98 (0.72–1.34)	0.9
Q4 (≥4.7)	0.90 (0.81–1.01)	0.08	0.85 (0.62–1.17)	0.32
Fasting blood sugar, mg/dL				
Q1 (<83)	1.00 [Reference]		1.00 [Reference]	
Q2 (83–87)	1.01 (0.83–1.23)	0.9	0.76 (0.52–1.11)	0.16
Q3 (88–95)	0.94 (0.82–1.08)	0.41	0.98 (0.69–1.38)	0.89
Q4 (≥96)	0.85 (0.73–0.98)	0.02	1.23 (0.90–1.67)	0.20
Total cholesterol, mg/dL				
Q1 (<169)	1.00 [Reference]		1.00 [Reference]	
Q2 (169–192)	1.09 (0.93–1.27)	0.28	0.55 (0.39–0.78)	<0.001
Q3 (193–217)	1.14 (0.94–1.39)	0.19	0.70 (0.45–1.08)	0.11
Q4 (≥218)	1.15 (0.90–1.48)	0.26	0.97 (0.57–1.66)	0.92
Triglyceride, mg/dL				
Q1 (<66)	1.00 [Reference]		1.00 [Reference]	
Q2 (66–95)	1.09 (0.94–1.26)	0.27	1.04 (0.77–1.39)	0.82
Q3 (96–145)	1.25 (1.08–1.44)	0.003	0.85 (0.61–1.17)	0.30
Q4 (≥146)	1.10 (0.92–1.30)	0.30	0.63 (0.42–0.94)	0.02
High-density lipoprotein, mg/dL				
Q1 (<48)	1.00 [Reference]		1.00 [Reference]	
Q2 (48–56)	0.83 (0.74–0.93)	0.001	0.96 (0.70–1.32)	0.82
Q3 (57–66)	0.71 (0.62–0.81)	<0.001	1.15 (0.83–1.58)	0.41
Q4 (≥67)	0.74 (0.63–0.85)	<0.001	1.09 (0.76–1.57)	0.64
Low-density lipoprotein, mg/dL				
Q1 (<90.8)	1.00 [Reference]		1.00 [Reference]	
Q2 (90.8–110)	0.92 (0.80–1.06)	0.26	0.78 (0.56–1.09)	0.14
Q3 (111–132)	0.86 (0.72–1.03)	0.10	0.71 (0.46–1.09)	0.11
Q4 (≥133)	0.89 (0.71–1.11)	0.30	0.58 (0.34–0.97)	0.04
Uric acid, mg/dL				
Q1 (<4.5)	1.00 [Reference]		1.00 [Reference]	
Q2 (4.5–5.3)	1.13 (0.98–1.30)	0.10	0.77 (0.56–1.06)	0.11
Q3 (5.4–6.4)	1.30 (1.14–1.49)	<0.001	0.94 (0.69–1.26)	0.66
Q4 (≥6.5)	1.53 (1.34–1.75)	<0.001	0.83 (0.60–1.15)	0.26
Proteinuria				
Grade 0	1.00 [Reference]		1.00 [Reference]	
Grade 1	1.32 (1.09–1.60)	0.005	0.95 (0.51–1.77)	0.88
Grade 2	1.25 (1.08–1.45)	0.003	1.08 (0.72–1.63)	0.70
≥Grade 3	1.69 (1.48–1.94)	<0.001	1.01 (0.61–1.69)	0.96
eGFR, ml/min/1.73 m^2^				
Q1 (<76.0)	1.00 [Reference]		1.00 [Reference]	
Q2 (76–87.4)	0.25 (0.22–0.28)	<0.001	1.28 (0.98–1.66)	0.07
Q3 (87.5–100.4)	0.12 (0.10–0.15)	<0.001	1.41 (1.05–1.91)	0.02
Q4 (≥100.5)	0.15 (0.11–0.19)	<0.001	2.12 (1.46–3.07)	<0.001

CI, confidence interval; Q, quartile.

The estimated glomerular filtration rate value was calculated by the CKD-EPI equation. Due to incomplete data of predictors, only 34,858 (75.3%) records were used for model development.

The illness-death model assuming the baseline hazard function was fitted Weibull distribution with shape: 5.7 and scale: 0.01 for transiting to eGFR <60 mL/min/1.73 m^2^, with shape: 6.8 and scale: 0.01 for transiting to death, and with shape: 7.7 and scale: 0.01 for transiting to death after eGFR state transition.

All the above factors are forced into the model, and a p-value <0.05 is considered statistical significance.

### Factors associated with late-phase eGFR state and death transitions

Patients with eGFR 59–30 mL/min/1.73 m^2^ were followed for an average of 7.7 years (IQR, 5.6–8.4), and a median-censored time of 3.9 years was recorded (IQR, 2.1–5.8). The significant independent factors for late-phase CKD state transition were male, DM, quartile of hemoglobin, quartile of fasting blood sugar, quartile of triglyceride, quartile of LDL, grade of proteinuria, and eGFR quartile (Table 3). However, only being a smoker, a low quartile of albumin, and a high grade of proteinuria were significantly associated with an excessive risk of direct transiting to death in patients with eGFR 59–30 mL/min/1.73 m^2^. The hazard functions for eGFR state and death transitions that developed based on selected covariates are displayed in Supplementary material.

**Table 3:** Factors associated with transiting to estimated glomerular filtration rate (eGFR) <30 mL/min/1.73 m^2^ or death in the cohort with baseline eGFR 59–30 mL/min/1.73 m^2^.

	**Transition to eGFR**<**30 mL/min/1.73 m**^**2**^		**Transition to death**	
	**Hazard ratio (95% CI)**	***p*-value**	**Hazard ratio (95% CI)**	***p*-value**
Sex				
Female	1.00 [Reference]		1.00 [Reference]	
Male	1.80 (1.29–2.53)	<0.001	1.24 (0.90–1.72)	0.19
Diabetes mellitus				
None	1.00 [Reference]		1.00 [Reference]	
Yes	1.52 (1.09–2.13)	0.01	1.02 (0.67–1.57)	0.9
Coronary artery disease				
None	1.00 [Reference]		1.00 [Reference]	
Yes	1.00 (0.86–1.16)	0.9	1.23 (0.92–1.64)	0.16
Smoker				
None	1.00 [Reference]		1.00 [Reference]	
Yes	0.98 (0.70–1.36)	0.9	1.39 (1.03–1.88)	0.03
Alcohol drinker				
None	1.00 [Reference]		1.00 [Reference]	
Yes	1.06 (0.74–1.52)	0.74	1.08 (0.78–1.49)	0.64
Regular exerciser				
None	1.00 [Reference]		1.00 [Reference]	
Yes	0.91 (0.69–1.21)	0.52	0.85 (0.65–1.11)	0.24
Metabolic syndrome				
None	1.00 [Reference]		1.00 [Reference]	
Yes	1.01 (0.84–1.21)	0.9	0.91 (0.78–1.05)	0.20
Hemoglobin, g/dL				
Q1 (<12.6)	1.00 [Reference]		1.00 [Reference]	
Q2 (12.6–13.6)	0.73 (0.52–1.03)	0.07	0.93 (0.65–1.34)	0.70
Q3 (13.7–14.6)	0.54 (0.38–0.79)	0.001	0.93 (0.65–1.32)	0.67
Q4 (≥14.7)	0.47 (0.31–0.70)	<0.001	0.90 (0.60–1.34)	0.60
Albumin, mg/dL				
Q1 (<4.2)	1.00 [Reference]		1.00 [Reference]	
Q2 (4.2–4.3)	0.95 (0.65–1.396)	0.79	0.79 (0.56–1.11)	0.17
Q3 (4.4–4.6)	1.06 (0.75–1.49)	0.74	0.79 (0.57–1.11)	0.17
Q4 (≥4.7)	0.86 (0.60–1.24)	0.43	0.68 (0.48–0.96)	0.03
Fasting blood sugar, mg/dL				
Q1 (<85)	1.00 [Reference]		1.00 [Reference]	
Q2 (85–92)	0.64 (0.43–0.94)	0.02	0.95 (0.67–1.34)	0.77
Q3 (93–103)	0.72 (0.49–1.08)	0.11	1.09 (0.75–1.58)	0.66
Q4 (≥104)	1.03 (0.68–1.57)	0.89	1.37 (0.91–2.07)	0.13
Total cholesterol, mg/dL				
Q1 (<180)	1.00 [Reference]		1.00 [Reference]	
Q2 (180–204)	1.17 (0.73–1.86)	0.52	0.85 (0.55–1.31)	0.45
Q3 (205–233)	0.65 (0.34–1.23)	0.19	0.79 (0.44–1.43)	0.43
Q4 (≥233)	0.77 (0.35–1.71)	0.52	0.47 (0.21–1.06)	0.07
Triglyceride, mg/dL				
Q1 (<88)	1.00 [Reference]		1.00 [Reference]	
Q2 (88–122)	1.10 (0.71–1.71)	0.67	1.16 (0.83–1.62)	0.39
Q3 (123–178)	1.47 (0.95–2.03)	0.09	1.16 (0.79–1.70)	0.46
Q4 (≥179)	1.75 (1.03–2.99)	0.04	1.39 (0.86–2.23)	0.18
High-density lipoprotein, mg/dL
Q1 (<47)	1.00 [Reference]		1.00 [Reference]	
Q2 (47–55)	0.75 (0.52–1.06)	0.11	0.85 (0.59–1.23)	0.39
Q3 (56–65)	0.66 (0.44–1.00)	0.05	0.94 (0.63–1.41)	0.78
Q4 (≥66)	0.77 (0.47–1.26)	0.30	1.10 (0.69–1.75)	0.68
Low-density lipoprotein, mg/dL				
Q1 (<97.4)	1.00 [Reference]		1.00 [Reference]	
Q2 (97.4–120.3)	1.23 (0.78–1.93)	0.38	0.91 (0.60–1.37)	0.64
Q3 (120.4–142)	1.97 (1.10–3.51)	0.02	0.95 (0.54–1.66)	0.85
Q4 (≥143)	1.71 (0.83–3.55)	0.15	1.44 (0.70–2.96)	0.33
Uric acid, mg/dL				
Q1 (<5.4)	1.00 [Reference]		1.00 [Reference]	
Q2 (5.4–6.4)	1.30 (0.84–2.00)	0.24	1.14 (0.80–1.62)	0.47
Q3 (6.5–7.8)	1.33 (0.87–2.02)	0.19	0.97 (0.67–1.40)	0.87
Q4 (≥7.9)	1.42 (0.94–2.15)	0.10	1.28 (0.88–1.85)	0.19
Proteinuria				
Grade 0	1.00 [Reference]		1.00 [Reference]	
Grade 1	1.61 (0.92–2.82)	0.1	1.22 (0.69–2.14)	0.50
Grade 2	1.61 (1.08–2.04)	0.02	1.63 (1.16–2.30)	0.005
≥Grade 3	3.60 (2.69–4.80)	<0.01	1.11 (0.75–1.63)	0.61
eGFR, ml/min/1.73 m^2^				
Q1 (<47.3)	1.00 [Reference]		1.00 [Reference]	
Q2 (47.3–53.0)	0.32 (0.23–0.43)	<0.01	0.95 (0.66–1.36)	0.78
Q3 (53.1–56.8)	0.14 (0.08–0.22)	<0.01	1.03 (0.72–1.46)	0.88
Q4 (≥56.9)	0.07 (0.04–0.13)	<0.01	0.89 (0.60–1.32)	0.56

CI, confidence interval; Q, quartile.

The estimated glomerular filtration rate value was calculated by the CKD-EPI equation.

The illness-death model assuming the baseline hazard function was fitted Weibull distribution with shape: 1.9 and scale: 0.01 for transiting to eGFR <30 mL/min/1.73 m^2^, with shape: 8.1 and scale: 0.01 for transiting to death, and with shape: 8.3 and scale: 0.01 for transiting to death after eGFR state transition.

All the above factors are forced into the model, and a p-value <0.05 is considered statistical significance.

### Model predictions

**Figure 3:**
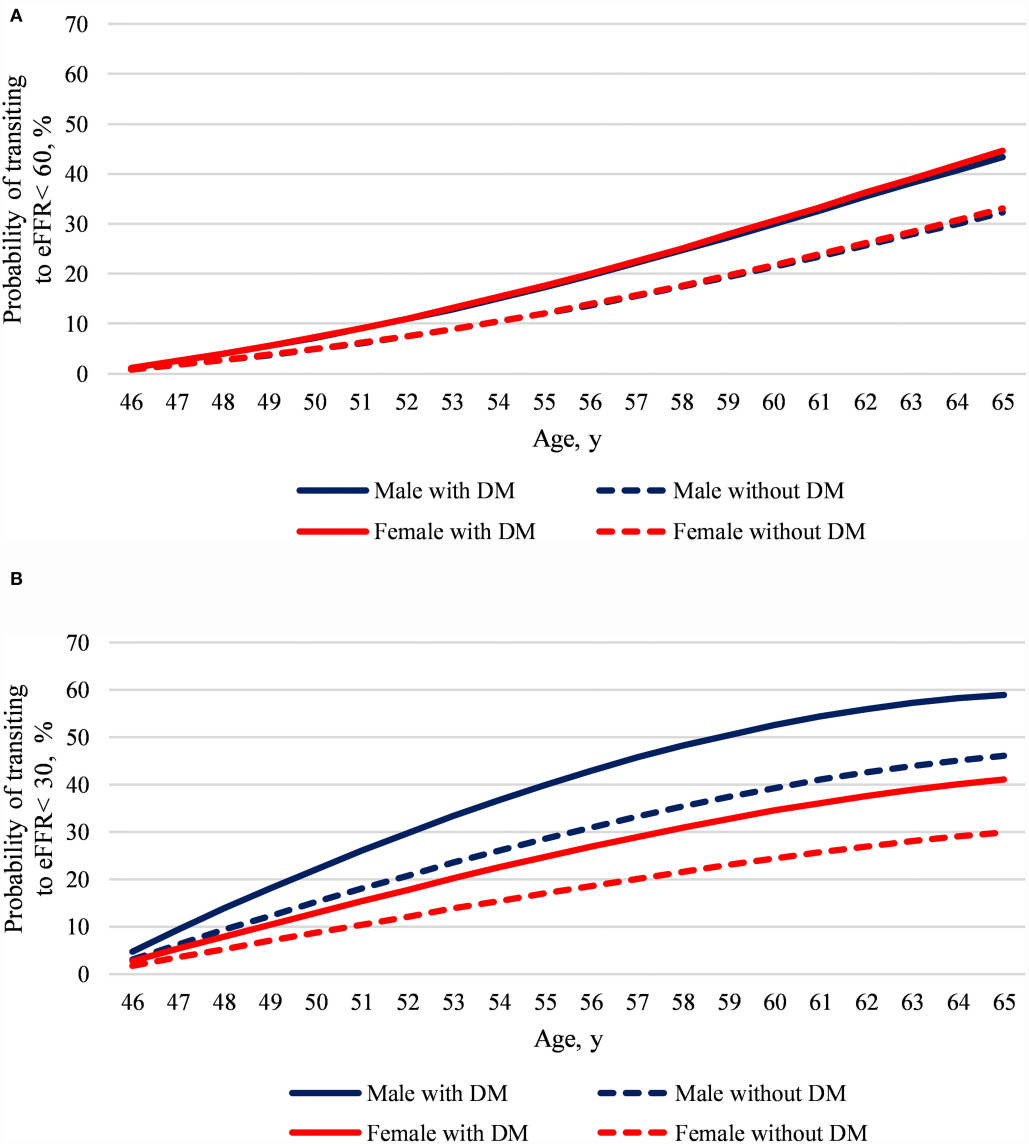
The model predicted age 46–65 CKD state transition risks at age 45 by sex and diabetes mellitus. **(A)** Predicted eGFR <60 transition risk and **(B)** predicted eGFR <30 transition risk. We set the reference values for factors other than sex and diabetes in the prediction models, said without coronary artery disease, selected life habitats, metabolic syndrome, and the lowest quartile of all baseline laboratory data. Despite the large study sample size and a median 7-year follow-up, the predicted risk may lose accuracy when it is extrapolated for a long period of time.

The developed model predicted the risks of defined eGFR state transition at age 46–65 in participants aged 45 and free for the state. The early-phase eGFR state transition model represents that regardless of which sex, DM compared with non-DM added a 0.3-time risk to transit to eGFR <60 in the next 20 years (Figure 3A). However, both sex and DM impact eGFR <30 transition risks. The late-phase eGFR state transition model displays that males with DM show a substantially doubled eGFR <30 transition risk in the next 20 years than females without DM (Figure 3B).

## Discussion

This community-based study is one of the few studies that quantified transition intensity for participants with different eGFR states and clarified factors influencing state transitions after accounting for competing risks of death. Our findings revealed that DM, fasting blood sugar, triglyceride, proteinuria grade, and eGFR were associated with early- and late-phase eGFR state transitions. However, uric acid and HDL were only linked to the early-phase eGFR state transition, whereas male, hemoglobin, and LDL were only related to the late-phase eGFR state transition. In addition, the developed models provide precisely eGFR state risk quantification at various ages by inputting participants' characteristics.

The primary tasks advocated by the Kidney Disease Outcomes Quality Initiative clinical guidelines are slow progression and avoiding immature death (23). Although the factors affecting clinical renal function deterioration have been identified during past decades, only a few pieces of evidence from community residents are available (24). The results of the current study show varying age-specific baseline hazard rates of the eGFR state and death transitions in community residences. The findings could be used to arrange different CKD screening activities for risky populations, such as shortening screening intervals and adopting more accurate tests (e.g., cystatin c and urine albumin creatine ratio). Further actions are needed to develop more precise CKD screening and referral policies based on the age-specific transition risks.

DM in a community is the main attributor for both eGFR state transitions. Routinely performing screenings by urinary and serum creatinine tests is the standard procedure for detecting early CKD transition. Although a recent study reported a dramatically improving trend in annual proportion of ≥ one-time serum creatinine or microalbuminuria tests in DM care (25), optimal screening intervals of renal function are still unknown and worthy of further study. Previous studies strongly support the finding that MetS is a crucial risk factor only for the early phase of renal function decline. Metabolic disorders are prevalent globally because of unhealthy lifestyles, such as increasing fast-food consumption and physical inactivity (26). Further research exploring the effects of correcting unhealthy lifestyles on the risk of proteinuria and early-phase renal function progression is wanted.

The current study showed the dose-response association between uric acid levels and risks of CKD state transition, especially in early CKD transition. It is worthy to notice that even high uric acid levels under therapeutic criteria could elevate the risk of CKD transition. High uric acid may result in arteriolosclerosis, glomerular injury, and tubulointerstitial fibrosis, either directly triggering urate crystals or indirectly causing vascular injuries (27). One systemic review study supported that improving uric acid abnormality by uric acid-lowering therapy would prevent kidney function decline in patients with CKD (28). In addition, our findings put insights into whether maintaining uric acid lower than the therapeutic standard would delay CKD progression.

It is not surprising that subjects with more severe proteinuria and lower eGFR levels at baseline were associated with high-risk CKD state transition. Our observations suggest that subjects maintain a higher level of eGFR, and controlling urine protein in a normal range is more critical in the late- than early-phase CKD. Although the mechanisms are unknown, they may associate with a non-linear decline of the renal reserve capacity from healthy to different CKD states (29). Contrary to intuition, we found that higher eGFR levels were associated with a higher hazard of directly transiting to death from eGFR ≥60 mL/min/1.73 m^2^. The findings may be attributed to comparing the transition hazard to eGFR <60 under the competing risk model structure, which could not be directly explained as an overall mortality hazard. However, it also substantially reflects the importance of applying a competing risk model structure rather than standard survival models when one study would explore factors influencing early-phase CKD transition. Male and low hemoglobin levels were two determinants for increasing state transition intensities only in patients whose eGFR was 59–30 ml/min/1.73 m^2^. Anemia is one of the primary complications in patients with late CKD stage (30). Recent international real-world evidence supports our findings that a higher hemoglobin level could benefit renal function progression and survival (31). Notably, our study repeals that sex is not associated with early-phase CKD transition, which was different from most previous observations but similar to one study that was analyzed using the illness-death model (32, 33). One possible explanation is the illness-death model incorporates interval-censored information, and death after CKD state transition makes our estimation less-biased. Our results of further analysis by the cause-specific hazard model, males had both higher hazards transiting to eGFR <60 (HR: 1.14, 95% CI: 1.02–1.28) and death (HR: 1.85, 95% CI: 1.47–2.34) than the females (data not shown), support this viewpoint. However, the mechanisms of sex disparity in different phases of CKD progression and therapy are complex and usually involved in biological, cultural, and socioeconomic dimensions (33), which are beyond the scope of our study. Therefore, more research is needed to explore the effects of sex-specific hormones, behaviors, and social–cultural on these discrepancies.

The roles of lipid profiles on CKD progression have not been well-established. Similar to the conclusion from a recent review, we did not find a consistent relationship between cholesterol on risks of transition in different CKD states (34). In this study, HDL plays a more significant role in early CKD state transition, whereas higher LDL levels were associated with a more transition risk to the advanced CKD stage. Given that both cholesterols have several hundred specials and may carry different proteins, the only quantifying cholesterol might not reflect the variety of biological effects of heterogeneous HDL and LDL on CKD progression (35). Therefore, exploring functional components of both cholesterols on CKD progression in great detail and evaluating their usability in the future are suggested.

This study had several strengths. First, it examined the changes in CKD states in a large cohort of community individuals over a long period, which assessed a sufficient number of desired events. Second, a multistate transition model can offer more holistic details about the influence of risk factors on renal function decline state. Third, using an illness-death model with Weibull distribution can estimate the absolute risk of CKD state transition. However, some limitations should be appropriately declared. First, we used only one laboratory result to determine the participant's eGFR state, which may not be accurate. Then, this study did not collect medications and behaviors, which may affect the effect estimation of laboratory levels. Third, the dynamic and multidimensional observed states make it challenging to evaluate the prediction performance of the developed models. However, all confidence regions for the model parameters generated by a simulation-based approach are located within a reasonable range, which reflects that our results should meet in line with practical reliability. Fourth, although our parametric model makes it possible to do any prediction even beyond the following-up time, one may apply it in line with caution. Finally, current findings were observed in the Asia population, limiting the generalization to other races.

## Conclusion

We documented independent factors of renal progression in a three-state transition model to classify the risks for community patients with different levels of eGFR. Moreover, we successfully developed two absolute risk functions for predicting renal function state transition. This information is beneficial for arranging more appropriate screening actions for participants with different risk characteristics of renal function state transitions and alerting physicians to early transfer the risky patients to receive more appropriate CKD management and care. The predicted probabilities by the developed models were particularly useful for high-risk screening attendees by arranging optimal screening intervals, avoiding substances-induced renal damage, and managing their chronic personal conditions.

## Data availability statement

The datasets presented in this article are not readily available because these data only could be analyzed under the government's regulations. Requests to access the datasets should be directed to https://chmuseum.klchb.gov.tw/default_2019.aspx.

## Ethics statement

The studies involving human participants were reviewed and approved by Ethical Committee of Health Bureau of Keelung City. The participants provided their written informed consent to participate in this study. All study procedures followed the principles of the Declaration of Helsinki.

## Author contributions

M-HT and M-YL: manuscript drafting, had full access to all data in the study and take responsibility for data integrity and accuracy of the data analysis, and administrative, technical, or material support. C-YH, AY, TC, and SC: concept and design. M-HT, C-YH, AY, TC, and SC: acquisition, analysis, or interpretation of data. TC and S-JH: critical revision of the manuscript for important intellectual content. M-YL: statistical analysis. All authors contributed to the article and approved the submitted version.

## Funding

We would like to thank the Taiwan Ministry of Science and Technology (Grant Number: MOST-108-2314-B-037-110) for supporting this work. The funders had no role in the design and conduct of the study, collection, management, analysis, and interpretation of the data, and preparation, review, or approval of the manuscript.

## Conflict of interest

The authors declare that the research was conducted in the absence of any commercial or financial relationships that could be construed as a potential conflict of interest.

## Publisher's note

All claims expressed in this article are solely those of the authors and do not necessarily represent those of their affiliated organizations, or those of the publisher, the editors and the reviewers. Any product that may be evaluated in this article, or claim that may be made by its manufacturer, is not guaranteed or endorsed by the publisher.

## Abbreviations

CKD: chronic kidney disease
CAD: coronary artery disease
CIs: confidence intervals
DM: diabetes mellitus
eGFR: estimated glomerular filtration rate
ESKD: end-stage kidney disease
HDL: high-density lipoprotein cholesterol
HR: hazard ratio
IQR: interquartile range
KCIS: Keelung Community-based Integrated Screening
LDL: low-density lipoprotein cholesterol
MetS: metabolic syndrome.
