# Supplementary material for: Factors associated with renal function state transitions: A population-based community survey in Taiwan
Source: Front Public Health. 2022 Sep 8;10:930798. doi: 10.3389/fpubh.2022.930798 (PMC9493090; doi:10.3389/fpubh.2022.930798)
Supplement: Supplementary file 1 [file Data_Sheet_1.docx]

***Hazard functions of early-phase eGFR state and death transitions***

The hazard function for Weibull distribution could be represented as $h\left( t \right)=\lambda\gamma t^{\gamma-1}$. Where the λ parameter determines the scale and $\gamma$ parameter determines the shape for the distribution. The transition intensities in the illness-death model could be represented by: $H_{ij}\left( t \right)=H_{0,ij}\left( t \right)exp\left\{ \beta_{ij}^{T}Z_{ijk} \right\}, ij\in\left\{ 01,02,12 \right\}$

Here $H_{0,ij}\left( t \right)$ are the baseline transition intensities, $Z_{ijk}$are subject *k*’s covariate vectors and $\beta_{ij}$ are vectors of regression parameters for transition *i* → *j*.^1^

The time-dependent transition probability could be displayed by:

$$\mathrm{tp}\left( t \right)=1-exp\left\{ H\left( t-\mu\right)-H\left( t \right) \right\}$$

$$=1-exp\left\{ \lambda\left( t-\mu\right)^{\gamma}-\lambda\left( t \right)^{\gamma} \right\}$$

$$=1-exp\left\{ \lambda\left[ \left( t-\mu\right)^{\gamma}-\left( t \right)^{\gamma} \right] \right\}$$

Where H(t) is the cumulative hazard rate.^2^ The parameters in the Weibull models are with shape parameterγ: 5.7 and scale parameter λ: 0.01 for transiting to estimated glomerular filtration rate (eGFR) <60 mL/min/1.73 m^2^, with shape parameterγ: 6.8 and scale parameter λ: 0.01 for transiting to death, and with shape parameterγ: 7.7 and scale parameter λ: 0.01 for transiting to death after eGFR state transition. Two risk prediction functions based on the factors listed in Table 2 are shown below:

1. Hazard function for eGFR<60 state transition from eGFR≥60 state$=h_{0}\left( t \right)Exp\left\{ -0.0070\times\left( Male \right)+0.4039\times\left( Diabetes mellitus \right)+0.0844\times\left( Cardiovascular disease \right)+0.0422\times\left( Smoker \right)+0.0084\times\left( Alcohol drinker \right)+0.1080\times\left( Betel nut chewer \right)+0.0233\times\left( Regular exerciser \right)+0.1119\times\left( Metabolic syndrome score \right)-0.0015\times\left( Dummy 1 for quartile of hemoglobin \right)-0.0672\times\left( Dummy 2 for quartile of hemoglobin \right)-0.0041\times\left( Dummy 3 for quartile of hemoglobin \right)+0.0256\times\left( Dummy 1 for quartile of albumin \right)+0.0213\times\left( Dummy 2 for quartile of albumin \right)-0.1017\times\left( Dummy 3 for quartile of albumin \right)+0.0092\times\left( Dummy 1 for quartile of fasting blood sugar \right)-0.0582\times\left( Dummy 2 for quartile of fasting blood sugar \right)-0.1662\times\left( Dummy 3 for quartile of fasting blood sugar \right)+0.0857\times\left( Dummy 1 for quartile of total cholesterol \right)+0.1313\times\left( Dummy 2 for quartile of total cholesterol \right)+0.1439\times\left( Dummy 3 for quartile of total cholesterol \right)+0.0820\times\left( Dummy 1 for quartile of triglyceride \right)+0.2226\times\left( Dummy 2 for quartile of triglyceride \right)+0.0915\times\left( Dummy 3 for quartile of triglyceride \right)-0.1887\times\left( Dummy 1 for quartile of high-density lipoprotein cholesterol \right)-0.3467\times\left( Dummy 2 for quartile of high-density lipoprotein cholesterol \right)-0.3098\times\left( Dummy 3 for quartile of high-density lipoprotein cholesterol \right)-0.0837\times\left( Dummy 1 for quartile of low-density lipoprotein cholesterol \right)-0.1514\times\left( Dummy 2 for quartile of low-density lipoprotein cholesterol \right)-0.1162\times\left( Dummy 3 for quartile of low-density lipoprotein cholesterol \right)+0.1183\times\left( Dummy 1 for quartile of uric acid \right)+0.2652\times\left( Dummy 2 for quartile of uric acid \right)+0.4262\times\left( Dummy 3 for quartile of uric acid \right)-1.3758\times\left( Dummy 1 for quartile of estimated glomerular filtration rate \right)-2.1193\times\left( Dummy 2 for quartile of estimated glomerular filtration rate \right)-1.9233\times\left( Dummy 3 for quartile of estimated glomerular filtration rate \right)+0.2757\times(Dummy 1 for grade of proteinuria)+0.2241\times(Dummy 2 for grade of proteinuria)+0.5273\times(Dummy 3 for grade of proteinuria) \right\}$.
2. Hazard function for death transition from eGFR≥60 state$=h_{0}\left( t \right)Exp\left\{ 0.6864\times\left( Male \right)+0.2658\times\left( Diabetes mellitus \right)+0.4479\times\left( Cardiovascular disease \right)+0.6388\times\left( Smoker \right)+0.0798\times\left( Alcohol drinker \right)+0.3267\times\left( Betel nut chewer \right)-0.1788\times\left( Regular exerciser \right)+0.0013\times\left( Metabolic syndrome score \right)-0.2498\times\left( Dummy 1 for quartile of hemoglobin \right)-0.4335\times\left( Dummy 2 for quartile of hemoglobin \right)-0.4450\times\left( Dummy 3 for quartile of hemoglobin \right)+0.1031\times\left( Dummy 1 for quartile of albumin \right)-0.0177\times\left( Dummy 2 for quartile of albumin \right)-0.1586\times\left( Dummy 3 for quartile of albumin \right)-0.2739\times\left( Dummy 1 for quartile of fasting blood sugar \right)-0.0252\times\left( Dummy 2 for quartile of fasting blood sugar \right)+0.2043\times\left( Dummy 3 for quartile of fasting blood sugar \right)-0.5951\times\left( Dummy 1 for quartile of total cholesterol \right)-0.3610\times\left( Dummy 2 for quartile of total cholesterol \right)-0.0281\times\left( Dummy 3 for quartile of total cholesterol \right)+0.0346\times\left( Dummy 1 for quartile of triglyceride \right)-0.1687\times\left( Dummy 2 for quartile of triglyceride \right)-0.4638\times\left( Dummy 3 for quartile of triglyceride \right)-0.0367\times\left( Dummy 1 for quartile of high-density lipoprotein cholesterol \right)+0.1360\times\left( Dummy 2 for quartile of high-density lipoprotein cholesterol \right)+0.0883\times\left( Dummy 3 for quartile of high-density lipoprotein cholesterol \right)-0.2480\times\left( Dummy 1 for quartile of low-density lipoprotein cholesterol \right)-0.3488\times\left( Dummy 2 for quartile of low-density lipoprotein cholesterol \right)-0.5515\times\left( Dummy 3 for quartile of low-density lipoprotein cholesterol \right)-0.2621\times\left( Dummy 1 for quartile of uric acid \right)-0.0670\times\left( Dummy 2 for quartile of uric acid \right)-0.1837\times\left( Dummy 3 for quartile of uric acid \right)+0.2444\times\left( Dummy 1 for quartile of estimated glomerular filtration rate \right)+0.3454\times\left( Dummy 2 for quartile of estimated glomerular filtration rate \right)+0.7503\times\left( Dummy 3 for quartile of estimated glomerular filtration rate \right)-0.0487\times(Dummy 1 for grade of proteinuria)+0.0807\times(Dummy 2 for grade of proteinuria)+0.0143\times(Dummy 3 for grade of proteinuria) \right\}$

***Hazard functions of late-phase eGFR state and death transitions***

The parameters in the Weibull models are with shape parameterγ: 1.9 and scale parameter λ: 0.01 for transiting to eGFR <30 mL/min/1.73 m^2^, with shape parameterγ: 8.1 and scale parameter λ: 0.01 for transiting to death, and with shape parameterγ: 8.3 and scale parameter λ: 0.01 for transiting to death after eGFR state transition. Two risk prediction functions based on the factors listed in Table 3 are shown below:

1. Hazard function for eGFR<30 state transition from eGFR 59–30 state$=h_{0}\left( t \right)Exp\left\{ 0.5917\times\left( Male \right)+0.4203\times\left( Diabetes mellitus \right)-0.0012\times\left( Cardiovascular disease \right)-0.0251\times\left( Smoker \right)+0.0602\times\left( Alcohol drinker \right)-0.0921\times\left( Regular exerciser \right)+0.0053\times\left( Metabolic syndrome score \right)-0.3111\times\left( Dummy 1 for quartile of hemoglobin \right)-0.6043\times\left( Dummy 2 for quartile of hemoglobin \right)-0.7611\times\left( Dummy 3 for quartile of hemoglobin \right)-0.0514\times\left( Dummy 1 for quartile of albumin \right)+0.0571\times\left( Dummy 2 for quartile of albumin \right)-0.1468\times\left( Dummy 3 for quartile of albumin \right)-0.4535\times\left( Dummy 1 for quartile of fasting blood sugar \right)-0.3217\times\left( Dummy 2 for quartile of fasting blood sugar \right)-0.0294\times\left( Dummy 3 for quartile of fasting blood sugar \right)+0.1532\times\left( Dummy 1 for quartile of total cholesterol \right)-0.4290\times\left( Dummy 2 for quartile of total cholesterol \right)-0.2622\times\left( Dummy 3 for quartile of total cholesterol \right)+0.0951\times\left( Dummy 1 for quartile of triglyceride \right)+0.3880\times\left( Dummy 2 for quartile of triglyceride \right)+0.5594\times\left( Dummy 3 for quartile of triglyceride \right)-0.2931\times\left( Dummy 1 for quartile of high-density lipoprotein cholesterol \right)-0.4110\times\left( Dummy 2 for quartile of high-density lipoprotein cholesterol \right)-0.2602\times\left( Dummy 3 for quartile of high-density lipoprotein cholesterol \right)+0.2050\times\left( Dummy 1 for quartile of low-density lipoprotein cholesterol \right)+0.6771\times\left( Dummy 2 for quartile of low-density lipoprotein cholesterol \right)+0.5382\times\left( Dummy 3 for quartile of low-density lipoprotein cholesterol \right)+0.2589 \times\left( Dummy 1 for quartile of uric acid \right)+0.2824\times\left( Dummy 2 for quartile of uric acid \right)+0.3502\times\left( Dummy 3 for quartile of uric acid \right)-1.1552\times\left( Dummy 1 for quartile of estimated glomerular filtration rate \right)-1.9914\times\left( Dummy 2 for quartile of estimated glomerular filtration rate \right)-2.6510\times\left( Dummy 3 for quartile of estimated glomerular filtration rate \right)+0.4757\times(Dummy 1 for grade of proteinuria)+0.4741\times(Dummy 2 for grade of proteinuria)+1.2795\times(Dummy 3 for grade of proteinuria) \right\}$
2. Hazard function for death transition from eGFR 59–30 state$=h_{0}\left( t \right)Exp\left\{ 0.2176\times\left( Male \right)+0.0236\times\left( Diabetes mellitus \right)+0.2082\times\left( Cardiovascular disease \right)+0.3317\times\left( Smoker \right)+0.0781\times\left( Alcohol drinker \right)-0.1626\times\left( Regular exerciser \right)-0.0971\times\left( Metabolic syndrome score \right)-0.0693\times\left( Dummy 1 for quartile of hemoglobin \right)-0.0769\times\left( Dummy 2 for quartile of hemoglobin \right)-0.1063\times\left( Dummy 3 for quartile of hemoglobin \right)-0.2424 \times\left( Dummy 1 for quartile of albumin \right)-0.2327\times\left( Dummy 2 for quartile of albumin \right)-0.3812\times\left( Dummy 3 for quartile of albumin \right)-0.0528\times\left( Dummy 1 for quartile of fasting blood sugar \right)+0.0840\times\left( Dummy 2 for quartile of fasting blood sugar \right)+0.3174\times\left( Dummy 3 for quartile of fasting blood sugar \right)-0.1678\times\left( Dummy 1 for quartile of total cholesterol \right)-0.2373\times\left( Dummy 2 for quartile of total cholesterol \right)-0.7625\times\left( Dummy 3 for quartile of total cholesterol \right)+0.1474\times\left( Dummy 1 for quartile of triglyceride \right)+0.1462\times\left( Dummy 2 for quartile of triglyceride \right)+0.3257\times\left( Dummy 3 for quartile of triglyceride \right)-0.1615\times\left( Dummy 1 for quartile of high-density lipoprotein cholesterol \right)-0.0584\times\left( Dummy 2 for quartile of high-density lipoprotein cholesterol \right)+0.0963\times\left( Dummy 3 for quartile of high-density lipoprotein cholesterol \right)-0.0959\times\left( Dummy 1 for quartile of low-density lipoprotein cholesterol \right)-0.0559\times\left( Dummy 2 for quartile of low-density lipoprotein cholesterol \right)+0.3618 \times\left( Dummy 3 for quartile of low-density lipoprotein \right)+0.1302\times\left( Dummy 1 for quartile of uric acid \right)-0.0296\times\left( Dummy 2 for quartile of uric acid \right)+0.2463\times\left( Dummy 3 for quartile of uric acid \right)-0.0506\times\left( Dummy 1 for quartile of estimated glomerular filtration rate \right)+ 0.0272\times\left( Dummy 2 for quartile of estimated glomerular filtration rate \right)-0.1153\times\left( Dummy 3 for quartile of estimated glomerular filtration rate \right)+ 0.1954\times(Dummy 1 for grade of proteinuria)+0.4889\times(Dummy 2 for grade of proteinuria)+0.1010\times(Dummy 3 for grade of proteinuria) \right\}$

STROBE Statement—Checklist of items that should be included in reports of ***cohort studies***

|  | Item No | Recommendation | Page No |
| --- | --- | --- | --- |
| **Title and abstract** | 1 | (*a*) Indicate the study’s design with a commonly used term in the title or the abstract | Title on page 1. |
|  |  | (*b*) Provide in the abstract an informative and balanced summary of what was done and what was found | Abstract on page 3. |
| **Introduction** | | | |
| Background/rationale | 2 | Explain the scientific background and rationale for the investigation being reported | Introduction on page 5. |
| Objectives | 3 | State specific objectives, including any prespecified hypotheses | Introduction on pages 5-6. |
| **Methods** | | | |
| Study design | 4 | Present key elements of study design early in the paper | Methods (study design) on page 6. |
| Setting | 5 | Describe the setting, locations, and relevant dates, including periods of recruitment, exposure, follow-up, and data collection | Methods (study design, data collection  ) on pages 6-9. |
| Participants | 6 | (*a*) Give the eligibility criteria, and the sources and methods of selection of participants. Describe methods of follow-up | Methods (study design) on page 6. |
|  |  | (*b*) For matched studies, give matching criteria and number of exposed and unexposed | N/A. |
| Variables | 7 | Clearly define all outcomes, exposures, predictors, potential confounders, and effect modifiers. Give diagnostic criteria, if applicable | Methods (*data collection, Mets, State of renal function decline*) on pages 6-7. |
| Data sources/ measurement | 8* | For each variable of interest, give sources of data and details of methods of assessment (measurement). Describe comparability of assessment methods if there is more than one group | N/A |
| Bias | 9 | Describe any efforts to address potential sources of bias | Methods (*Statistical analysis*)  on pages 8-9. |
| Study size | 10 | Explain how the study size was arrived at | N/A |
| Quantitative variables | 11 | Explain how quantitative variables were handled in the analyses. If applicable, describe which groupings were chosen and why | Methods (*Statistical analysis*)  on pages 8-9. |
| Statistical methods | 12 | (*a*) Describe all statistical methods, including those used to control for confounding | Methods (*Statistical analysis*)  on pages 8-9. |
|  |  | (*b*) Describe any methods used to examine subgroups and interactions | None. |
|  |  | (*c*) Explain how missing data were addressed | None. |
|  |  | (*d*) If applicable, explain how loss to follow-up was addressed | N/A. |
|  |  | (*e*) Describe any sensitivity analyses | None. |
| **Results** |  |  |  |
| Participants | 13* | (a) Report numbers of individuals at each stage of study—eg numbers potentially eligible, examined for eligibility, confirmed eligible, included in the study, completing follow-up, and analysed | Results (*Study participant characteristics*) on page 10. |
|  |  | (b) Give reasons for non-participation at each stage | Results (*Study participant characteristics*) on page 10. |
|  |  | (c) Consider use of a flow diagram | Fig 1 |
| Descriptive data | 14* | (a) Give characteristics of study participants (eg demographic, clinical, social) and information on exposures and potential confounders | Table 1. |
|  |  | (b) Indicate number of participants with missing data for each variable of interest | N/A. |
|  |  | (c) Summarise follow-up time (eg, average and total amount) | Results (*Factors associated with early-phase eGFR state and death transitions, and* *Factors associated with late-phase eGFR state and death transitions* paragraphs) on pages 10-11. |
| Outcome data | 15* | Report numbers of outcome events or summary measures over time | Results (*Factors associated with early-phase eGFR state and death transitions, and* *Factors associated with late-phase eGFR state and death transitions* paragraphs) on pages 10-11. |
| Main results | 16 | (*a*) Give unadjusted estimates and, if applicable, confounder-adjusted estimates and their precision (eg, 95% confidence interval). Make clear which confounders were adjusted for and why they were included | Table 2&3. |
|  |  | (*b*) Report category boundaries when continuous variables were categorized | N/A. |
|  |  | (*c*) If relevant, consider translating estimates of relative risk into absolute risk for a meaningful time period | None |
| Other analyses | 17 | Report other analyses done—eg analyses of subgroups and interactions, and sensitivity analyses | Results (*Model predictions* paragraph*)* on page 11-12. |
| **Discussion** | | | |
| Key results | 18 | Summarise key results with reference to study objectives | Discussion on page 13. |
| Limitations | 19 | Discuss limitations of the study, taking into account sources of potential bias or imprecision. Discuss both direction and magnitude of any potential bias | Discussion on page 15. |
| Interpretation | 20 | Give a cautious overall interpretation of results considering objectives, limitations, multiplicity of analyses, results from similar studies, and other relevant evidence | Discussion on pages 13-15. |
| Generalisability | 21 | Discuss the generalisability (external validity) of the study results | Limitation on page 15. |
| **Other information** | | | |
| Funding | 22 | Give the source of funding and the role of the funders for the present study and, if applicable, for the original study on which the present article is based | Financial disclosure on page 16. |

*Give information separately for exposed and unexposed groups.

**Note:** An Explanation and Elaboration article discusses each checklist item and gives methodological background and published examples of transparent reporting. The STROBE checklist is best used in conjunction with this article (freely available on the Web sites of PLoS Medicine at http://www.plosmedicine.org/, Annals of Internal Medicine at http://www.annals.org/, and Epidemiology at http://www.epidem.com/). Information on the STROBE Initiative is available at <http://www.strobe-statement.org>.

Reference

**1.** Touraine C, Gerds TA, Joly P. The SmoothHazard package for R: Fitting regression models to interval-censored observations of illness-death models. *Copenhagen: University of Copenhagen, Department of Biostatistics (Research Report 13/12).* 2013.

**2.** Briggs A, Sculpher M, Claxton K. *Decision modelling for health economic evaluation*: Oup Oxford; 2006.
